# Hyperventilation and Hypoxia Hangover During Normobaric Hypoxia Training in Hawk Simulator

**DOI:** 10.3389/fphys.2022.942249

**Published:** 2022-07-13

**Authors:** Nikke Varis, Antti Leinonen, Kai Parkkola, Tuomo K. Leino

**Affiliations:** ^1^ Faculty of Medicine and Health Technology Tampere University, Tampere, Finland; ^2^ School of Medicine University of Eastern Finland, Kuopio, Finland; ^3^ National Defense University, Helsinki, Finland; ^4^ Aeromedical Centre Centre for Military Medicine, Helsinki, Finland

**Keywords:** hypoxia, hyperventilation, military aviation, flight simulation, ventilation, hypocapnia, carbon dioxide

## Abstract

**Introduction:** In military aviation during high-altitude operations, an oxygen or cabin pressure emergency can impair brain function and performance. There are variations in individuals’ physiological responses to low partial pressure of oxygen and hypoxia symptoms can vary from one exposure to another. The aim of this study was to evaluate how normobaric hypoxia (NH) affects pilots’ minute ventilation and 10 min afterwards on Instrument Landing System (ILS) flight performance in Hawk simulator during a tactical flight sortie.

**Methods:** Fifteen volunteer fighter pilots from the Finnish Air Force participated in this double blinded, placebo controlled and randomized study. The subjects performed three flights in a tactical Hawk simulator in a randomized order with full flight gear, regulators and masks on. In the middle of the flight without the subjects’ knowledge, 21% (control), 8% or 6% oxygen in nitrogen was turned on. Minute ventilation (VE) was measured before, during NH and after NH. Forehead peripheral oxygen saturation (SpO_2_), wireless ECG and subjective symptoms were documented. The flights were conducted so that both subjects and flight instructors were blinded to the gas mixture. The pilots performed tactical maneuvers at simulated altitude of 20,000 ft or 26,000 ft until they recognized the symptoms of hypoxia. Thereafter they performed hypoxia emergency procedures with 100% oxygen and returned to base (RTB). During the ILS approach, flight performance was evaluated.

**Results:** The mean VE increased during NH from 12.9 L/min (21% O2 on the control flight) to 17.8 L/min with 8% oxygen (*p* < 0.01), and to 21.0 L/min with 6% oxygen (*p* < 0.01). Ten minutes after combined hyperventilation and hypoxia, the ILS flight performance decreased from 4.4 (control flight) to 4.0 with 8% oxygen (*p* = 0.16) and to 3.2 with 6% oxygen (*p* < 0.01). A significant correlation (r = -0.472) was found between the subjects’ VE during 6% oxygen exposure and the ILS flight performance.

**Discussion:** Hyperventilation during NH has a long-lasting and dose-dependent effect on the pilot’s ILS flight performance, even though the hypoxia emergency procedures are executed 10 min earlier. Hyperventilation leads to body loss of carbon dioxide and hypocapnia which may even worsen the hypoxia hangover.

## Introduction

Despite the improvements of modern fighters and their equipment, physiological episodes (PEs) are reported by military pilots (Rice et al., 2019). PEs have occurred at alarming rates during the last decade. Problems were suspected to be related to the malfunction of the On-Board Oxygen Generation System (OBOGS) or loss of cabin pressurization leading to a low partial pressure of oxygen at high altitude. The latest reports have found that PEs are decreasing in number (Watters, 2020). The US Navy is also intensifying maintenance of environmental control system (ECS) and it has led to an 88% reduction in pressure related PEs in older generation F-18s (Pawlyk, 2020). Still the “smoking gun” of PEs has not been clearly identified. A recent study conducted of the United Kingdom Eurofighter fleet concluded that most in-flight symptoms reported were due to hyperventilation rather than hypoxia (Connolly et al., 2021).

Deprivation of oxygen causes increased ventilation (Bustamante-Sánchez et al., 2019). Symptoms of hypoxia in young pilots are usually shortness of breath, lightheadedness, pressure in the head, cognitive impairment, tingling and visual disturbance, and the symptoms are noticed to vary between individuals (Rice et al., 2019). Hypoxia causes cognitive deficits that may include longer reaction time, decision-making impairment and certain types of memory loss, and hyperventilation may confound these deficits (Petrassi et al., 2012). Hypoxia itself induces a transient cerebrovascular vasodilatation (Guadagno et al., 2011), but reflexive hyperventilation leading to hypocapnia results in vasoconstriction, which reduces the cerebral perfusion (Petrassi et al., 2012).

Only the partial pressure of inspired oxygen (PiO2) is equivalent between hypobaric hypoxia (HH) and normobaric hypoxia (NH). During short, less than 5 min exposure, to very low PiO2 simulating an altitude of 25,000 ft, physiological differences between HH and NH are apparent but minor (Richard & Koehle, 2012). However, a longer 30 min exposure to 18,045 ft with HH is reported to lead to more hypoxia symptoms compared to NH (Aebi et al., 2020).

When the body detects a lowered level of oxygen, the physiological response is to hyperventilate (Schoene, 2001). Westerman (2004) reported that during NH the minute ventilation of pilots increased from 6.7 to 16.4 L/min. In another study they concluded that the minute ventilation was raised from 11 to 16.5 L/min (Westerman et al., 2010). Uchida et al. (2020) reported a 10% increase in ventilation while subjects inspired hypoxic air, and that the changes were progressive. These changes went back to normal in the recovery phase. There are also hypoxic hypoxia studies that report no change in the ventilation rate or breathing frequency, but in these settings the level of exposure to hypoxia was mild since the peripheral oxygen saturation was around 90% (Savourey et al., 2003; Steinman et al., 2017). Reflexive hyperventilation has been found to be similar in hypobaric hypoxia and normobaric hypoxia (Loeppky et al., 1996). Arterial blood carbon dioxide partial pressure and/or alkalosis have negative effects on the cognitive performance (Leacy et al., 2019; Friend et al., 2019; van Dorp et al., 2007). This may be due to altered regional blood flow and oxygenation caused by hypocapnia.

Peripheral blood oxygen saturation (SpO2) is an estimate of the arterial blood oxygen partial pressure (SaO2). On the other hand, hyperventilation induced hypocapnia leads to respiratory alkalosis which shifts the SaO2-PiO2 curve leading to a non-linear decrease in SpO2 during hypoxia. As seen in previous studies (Malle et al., 2013), an SpO2 decrease during 6% NH is not linear including the flattening of SpO2 after 1 min of hypoxia. Compensatory mechanisms of the body are the reason for the non-linear decrease of SpO2 during the last phase of 6% NH exposure.

The most worrisome part of hypoxia exposure is the reduced ability to safely fly the aircraft. Steinman et al. (2017) reported that pilots’ flight performances worsened even at altitude levels of 15,000 ft, while flying in a simulator in a hypobaric chamber without supplementary oxygen. Nesthus et al. (1997) also noticed that there were more errors during approach and landing at altitudes as low as 8,000 ft. In our previous study NH resulted in a 25% reduction in ILS flight performance, even though hypoxia was treated with 100% emergency oxygen 10 min earlier (Varis et al., 2019). Recently, after 9.7% O2, delayed recovery has been documented using EEGs, reaction times and auditory processing (Blacker and McHail, 2021, 2022).

NH training in flight simulators is mandatory in the Finnish Air Force every 3 years to refresh the ability of fighter pilots to detect early hypoxic symptoms (Leinonen et al., 2021). Some pilots react to hypoxic exposure with rapid hyperventilation, and this phenomenon can affect their cognition and flight performance. The primary response to low partial pressure of oxygen may be related to hyperventilation and hypocapnia-induced (i.e., poikilocapnic hypoxia) decrement of cerebral blood flow leading to impaired cerebral oxygen saturation (ScO2) (Virués-Ortega et al., 2004).

A purpose of this study was to document individual minute ventilation response during normobaric hypoxia and analyze how combined hyperventilation and hypoxia affect ILS flight performance 10 min following exposure in Hawk simulator during tactical flight sortie.

## Methods

### Subjects

This study was performed in Fighter Squadron 41 (Tikkakoski, Finland). The study protocol followed the tenets of the Declaration of Helsinki, and it was approved by the Defense Command Finland (AQ24262, 2020) and the Ethics Committee of Tampere University Hospital (R18008, 2018). Fifteen qualified Hawk fighter pilots with an active flight status participated in this study. The study group were an average age of 24.6 years old (SD 0.51) and consisted of male subjects only, because no female pilots reported for hypoxia training during the study. The participants had an average of 246 (SD 8; range 231–265) total flight hours experience, within an average of 156 (SD 8; range 143–177) flight hours in a Hawk. They had all completed hypoxia training earlier in a hypobaric chamber and received theoretical refresher training on the subjective and objective signs of hypoxia.

### Equipment

**FIGURE 1:**
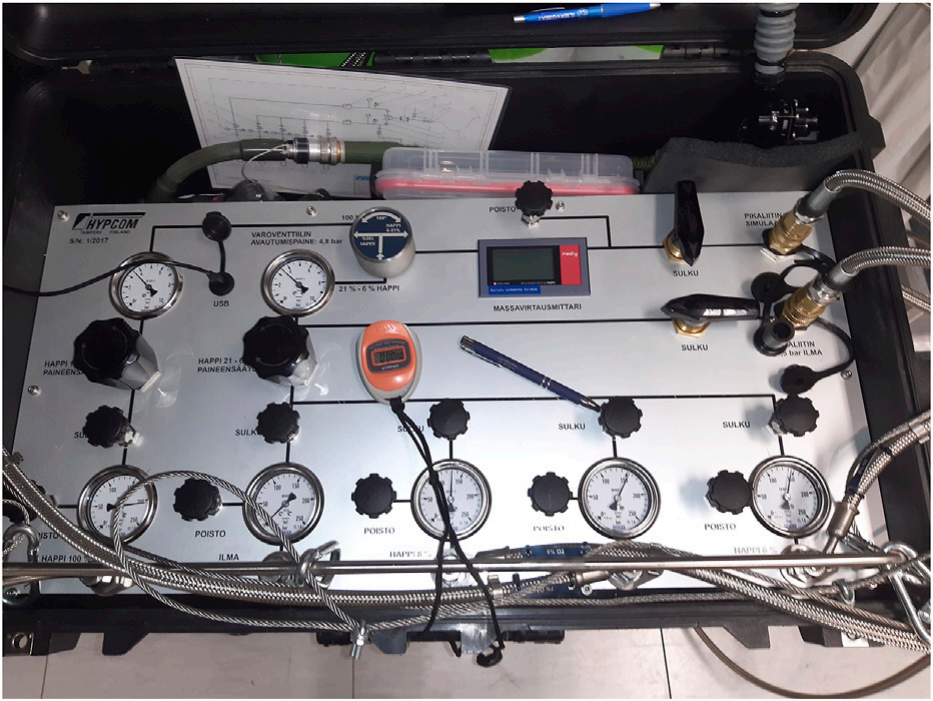
Hypoxic gas selection box (Hypcom, Tampere, Finland).

The hypoxia training was performed in a tactical Hawk Mk51A MLU flight simulator at an elevation of 140 m. The participants were equipped with full military flight gear, including a regulator, and oxygen mask. The forehead SpO2, VE and wireless ECG were continuously monitored during the experiment by a flight surgeon. SpO2, VE, and subjective symptoms were manually saved to a data sheet by an experienced flight nurse. The flight instructor and the flight surgeon had audio-visual access to the subjects *via* cameras, flight instruments and front sector screens. We commissioned four high-pressure cylinders with different concentrations of O_2_: 6%, 8%, 21% (equal to sea level) and 100% (emergency oxygen). These cylinders were connected to the simulator’s oxygen system and allowed the flight surgeon to manually change the selection for each subject with a gas selection box (Hypcom, Tampere, Finland) (Figure 1). In each flight in randomized order, the following concentrations of oxygen were used to induce hypoxia in a simulated high-altitude phase of flight under normobaric simulator conditions:

• 6% O_2_ to simulate physiological altitude of 7,900 m (25,919 ft)
• 8% O_2_ to simulate physiological altitude of 6,200 m (20,341 ft)
• 21% O_2_ as a blinded control

### Design

The study included three flights in a tactical Hawk simulator with the mask on, and a sudden onset of different oxygen concentrations (6%, 8%, and 21%) randomized by the flight surgeon. Both the subjects and the flight instructors were blinded to the gas mixture used during the flight. The RTB after hypoxia emergency procedures was evaluated by two experienced flight instructors (Patria Pilot Training, Tikkakoski, Finland). The evaluation was based on the standardized Finnish Air Force grading system for ILS flight performance found in the FINAF Hawk Standard Operations Manual, which has also been used in our previous study (Varis et al., 2019). The maximum performance score is 5 and the minimum is 1. The minute ventilation was measured for a 30 s period at three points: 1) “beginning” = the pilots were climbing towards the training area at low altitude, 2) “exposure” = starting 45 s after changing to hypoxic gas, and 3) “return” = 120 s after hypoxia emergency procedures and emergency descent during the RTB.

### Procedure

The study was conducted as part of the normal hypoxia training in the Finnish Air Force. Subjects were briefed to perform three flights, and in each one a gas exchange occurred. All the flights were performed on the same day with at least 60 min separation between the flights to ensure a wash out from previous exposure. In the tactical Hawk simulator, the weather conditions were a Runway Visual Range of 1,000 m, overcast at 300 ft, a wind of 4 knots and cloud top at 6,000 ft. After take-off from Jyväskylä airfield (EFJY), the pilots climbed to 20,000 or 25,000 ft and carried out tactical maneuvering including barrel-rolls and wingovers. During maneuvering the pilots were also given a mental workload by the Air Traffic Control, such as calculating new minimum fuel calculations (BINGO) for an alternative airfield or new lower limit on training area.

At the beginning of each flight, the subjects were given pressurized air (21% O2), but the flight surgeon switched to 21% (simulated switch), 8% or 6% oxygen during tactical maneuvering. The pilots continued the flight mission until they recognized hypoxia symptoms or there was a system warning (MASTER CAUTION and OXY light) and then executed hypoxia emergency procedures. The emergency procedures during the oxygen failure were: 1) emergency oxygen (100%) on, 2) oxygen main valve off, 3) emergency descent at 20° nose-down attitude and 4) transponder code 7700 (emergency squawk).

After the hypoxia emergency procedures, the pilots returned to the Jyväskylä airfield in instrument meteorological conditions (IMC) and used an RNAV approach technique. The RTB was made more difficult with a GPS malfunction and the pilots had to use non-gyro vectors and waterline Heads-Up Display mode during an ILS Z 30 approach. The ILS approach was evaluated with the instrument flight examination protocol from the final approach fix (FAF) to decision altitude (DA) as published previously (Varis et al., 2019).

### Statistical Analysis

A data analysis was performed using SPSS (IBM SPSS Statistics 25). The flying performance was graded on a non-linear scale of 1–5, with 1 being the worst and 5 being the best result. The score was calculated as the mean value from three subcategories: Loc, GS and alpha (Vi). Repeated measures analyses of variance (repANOVAs) were conducted to evaluate the change in ventilation during exposures to 8%, 6%, and 21% O2. The Bonferroni correction was applied to post hoc tests. The association between hypoxia and flight performance was calculated using the Friedman test because the flight performance values were not normally distributed. Post hoc analysis with Wilcoxon signed-rank tests was conducted with the Bonferroni correction applied. The data is presented as the mean + − the Standard Deviation (SD). The correlations between ventilation, oxygen saturation and flight performance during hypoxic RTB were evaluated using Pearson’s correlation coefficients. A *p*-value below 0.05 was deemed to be statistically significant.

## Results

**FIGURE 2:**
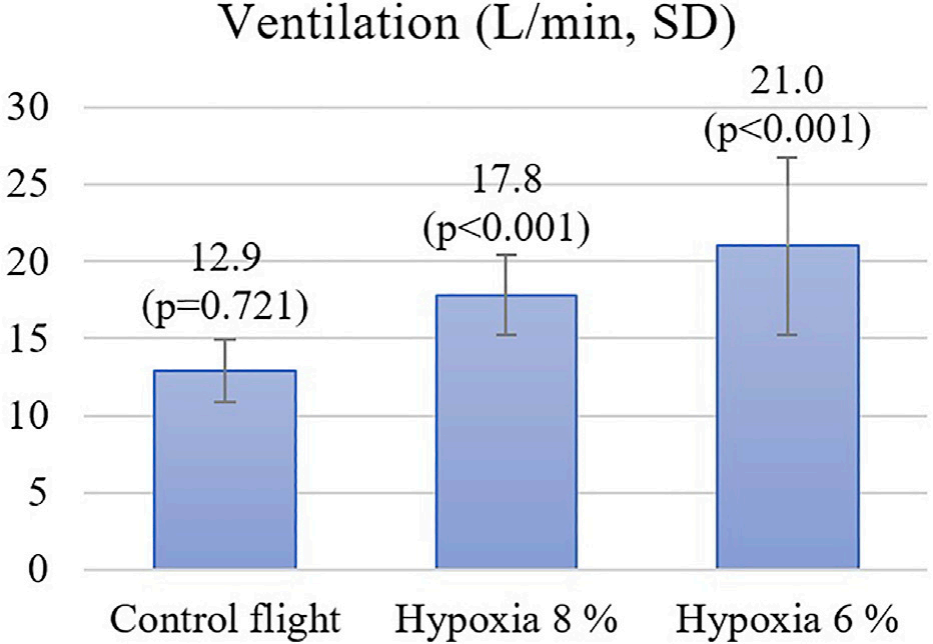
The minute ventilation during the exposure gas (21%, 8% or 6%).

**FIGURE 3:**
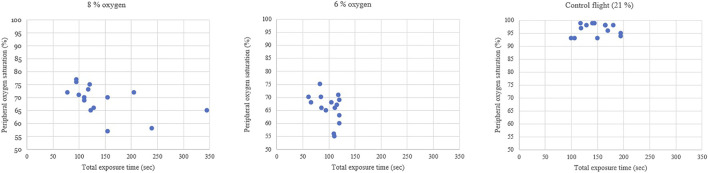
The peripheral oxygen saturation after hypoxia emergency procedures.

During the flight when pilots were exposed to 8% oxygen, they noticed symptoms of hypoxia on average 103 s after the hypoxic mixture was induced. Their mean SpO2 was at this point 74% and the heart rate increased from 93 to 112. The VE increased statistically significantly by 4.2 L/min, SD 2.3 (*p* < 0.001) from 13.6 L/min to 17.8 L/min (Figure 2). The pilots had executed all the hypoxia emergency procedures on average at 145 s (SpO_2_ of 69%) since the introduction of the hypoxic gas mixture (Figure 3). The oxygen dose when the exposure time was also counted was 62% lower compared to the control flight.

During the flight with a gas mixture of 6% oxygen, the pilots noticed their hypoxic symptoms on average after 75 s. The SpO_2_ readings decreased to 69% and the heart rate increased from 98 to 124. The VE increased statistically significantly by 7.6 L/min, SD 5.0 (*p* < 0.001) from 13.4 L/min to 21.0 L/min. During this flight, all the hypoxia emergency procedures were done on average 100 s since the introduction of the hypoxic gas (SpO_2_ of 66%). The oxygen dose when exposure time was also counted was 71% lower compared to the control flight.

During the blinded control flight with 21% oxygen, the SpO_2_ did not change and heart rate increased only from 94 to 104 during tactical maneuvering in the simulator. At the beginning of this flight, the VE was 12.8 L/min and during 21% O2 exposure it was 12.9 L/min, which was not statistically different from the starting value (*p* = 0.721). The exposure period of 100 s ended on MASTER CAUTION and OXY light, followed by emergency procedures.

**FIGURE 4:**
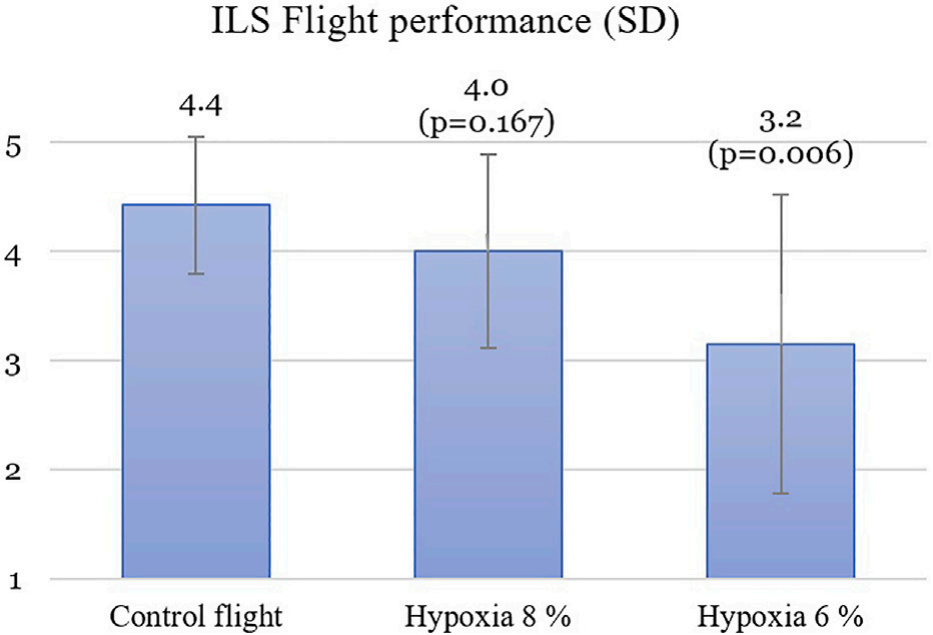
The Instrument landing system flight performance during the return to base.

During the control flight the pilots scored on average of 4.42 out of 5 points on the ILS flight performance. Ten minutes after hypoxia emergency procedures, the post-hypoxic scores for the ILS flight performance decreased. They were 4.00 points with 8% oxygen (Z -1.38; *p* = 0.167) and 3.16 points with 6% oxygen (Z -2.74; *p* = 0.006) (Figure 4).

**FIGURE 5:**
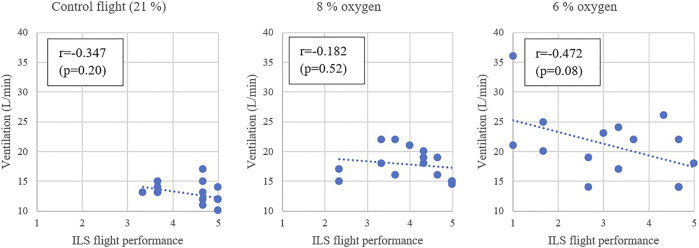
The minute ventilation during the exposure gas and the ILS flight performance during the RTB.

Correlations were calculated between the subjects’ VE and waterline ILS flight performance (Figure 5). For the flight with an 8% oxygen mixture the Pearson’s correlation to hyperventilation was −0.182 (very weak) and for the flight with 6% oxygen mixture the correlation was moderate at −0.472. Comparing the subjects’ ventilation rates in 8% and 6% oxygen, it was found that the Pearson’s correlation was 0.386, suggesting similarity of individual reflexive hyperventilation at different levels of hypoxia exposure.

A higher VE during hypoxia had a correlation with a higher SpO2 saturation at the end of the exposure with 6% oxygen (0.307). Additionally, a higher SpO2 saturation at the end of the hypoxic exposure had very strong correlation of −0.616 with a worsened ILS flight performance after a 6% oxygen exposure.

After each flight, the subjects were asked what hypoxia symptoms they had recognized. The most common symptoms were lightheadedness, visual impairment and dizziness (Table 1). One subject experienced symptoms during the 21% O2 flight and conducted the emergency procedures.

**TABLE 1:** Reported symptoms after hypoxia exposure.

	8 (%)hypoxia (*n* = 15)	6 (%)hypoxia (*n* = 15)
Lightheadedness	73%	47%
Visual impairment	47%	33%
Dizziness	33%	53%
Impaired cognition	33%	27%
Hot flushes	23%	20%
Shortness of breath	20%	27%
Feeling of pressure	20%	27%
Tingling of skin	20%	13%

## Discussion

During their first simulator hypoxia training, young Hawk military pilots recognized their hypoxia symptoms on average at an SpO2 saturation level of 69% during 6% oxygen exposure. This is a similar result compared to older pilots in the Finnish Air Force, with one study indicating SpO2 levels in older pilots of 77% (Varis et al., 2019) and another indicating 73% (Leinonen et al., 2021). SpO2 is known only very weakly to predict aspects such as working memory impairment (Malle et al., 2013). During normobaric hypoxia training, the exposure time is a more important parameter than SpO2, although the US Navy uses 60% SpO2 as an abort point. After the rapid development of hypoxia, hyperventilation and hypoxia already affect the pilots’ cognition and ability to perform hypoxia emergency procedures. From cognitive awareness at the point of hypoxia symptom recognition, it took 25–42 s to execute all the hypoxia emergency procedures. This highlights the importance of making an early decision to abort flight missions and illustrates the importance of the cognitive ability to change the mental focus from the operational flight task to emergency procedures creating a safety margin before the onset of more severe cognitive impairment (Johnston et al., 2012). For some pilots it was hard to recognize their individual hypoxia symptoms, for example, one of our subjects took 340 s to execute all the hypoxia emergency procedures. If a pilot does not recognize the symptoms early enough, there is a risk of loss of consciousness. In a recent study, 42% of student naval aviators could not recognize their hypoxia symptoms during their first hypoxia experience in a simulator (Rice et al., 2019).

The symptoms reported in this study, at least light-headedness, dizziness and tingling of skin are actually hyperventilation induced hypocapnia symptoms (Shaw et al., 2021). In the study by Westerman et al. (2010), the minute ventilation during 6% oxygen was 16.4 L/min and the hypoxia symptoms reported were: visual impairment 65%, autonomic symptoms 45%, neuromuscular symptoms 38%, headaches 37% and dizziness 21%. Thus, many of the symptoms recognized during hypoxia are likely due to hyperventilation. Large individual variations in the minute ventilation were also observed in our study. This can be one explanation why hypoxia symptoms in the same individuals can vary from one hypoxia training event to another. Pilots with a slow ventilation rate during hypoxia may lack all of the learned symptoms and have difficulties identifying hypoxia because of the lack of hyperventilation induced hypocapnia symptoms.

During hypoxia, the subjects’ heart rate increased to a level of 112 (8% of O2) and 124 (6% of O2) which is identical to previous study reports of heart rate of 120 during 6% O2 during hypoxia. During our control flight, tactical maneuvering at 25,000 ft resulted in an increased heart rate of 104. This mirrors the cognitive workload of military flying at the point when hypoxic conditions were introduced in our study. The cognitive workload rapidly affects cardiac parasympathetic control leading to decreased time between two successive R-waves of the QRS (Lahtinen et al., 2007). Cardiorespiratory upregulation during hypoxia, as seen with increased heart rate, also includes an increase in the cardiac output.

Westerman et al. (2010) reported a starting minute ventilation of 7 L/min. Our subjects used full fighter flight gear, pressure regulators and masks, resulting in a minute ventilation of 12 L/min in the same phase of flight. The VE increased significantly during hypoxic exposure and the increase was dose dependent in our study. More interestingly, hyperventilation during hypoxia had negative correlation to the ILS flight performance 10 min afterwards. Malle et al. (2013) have shown that the SpO2 will return to the baseline after hypoxia emergency procedures within 120 s but that the cognitive impairment and working memory are affected for much longer. Hypoxia induced hyperventilation will lead to hypocapnia, which causes cerebral vasoconstriction, and brain hypoperfusion (Gradwell & Rainford, 2016). 100% emergency oxygen after this may even worsen the brain recovery due to oxygen induced vasoconstriction of the cerebral arteries.

The ILS flight performance decreased in both of the hypoxic scenarios compared to the control flight. This confirms the hypoxia hangover findings from our previous study, in which the ILS flight performance decreased from 4.8 to 3.6 after three hypoxic set-ups in one flight (Varis et al., 2019). Earlier it was speculated that hypoxia hangover could be due to the cumulative effect of three hypoxic exposures, but in this study, the worsened ILS flight performance was shown in placebo controlled, randomized, double blind study protocol. Nevertheless, it must be acknowledged that symptoms in the second hypoxia exposure may have some cumulative effect from the previous exposure since they were conducted during the same day. Many studies have previously shown how cognition is affected during hypoxia. Hypoxia impairs the working memory, increases the reaction time and reduces executive functions (Malle et al., 2013; Neuhaus and Hinkelbein, 2014; Dart et al., 2017; Takács et al., 2017). Normobaric hypoxia also produces increases in the perceived workload (Stephens et al., 2017).

Our study confirms that normobaric hypoxia has a long-lasting effect on a pilot’s flight performance even when hypoxia emergency procedures are executed without delay 10 min earlier. Moreover, the present study suggests a dose-dependent effect of hyperventilation during hypoxia based on the ILS flight performance with 4.42 of 21% O2, 4.00 of 8% O2 and 3.6 of 6% O2. The reaction time and regional cerebral saturation do not return to baseline levels until 24 h following hypoxia exposure (Phillips et al., 2015). This is why the Finnish Air Force uses 1 day grounding after hypoxia training or in-flight hypoxia symptoms.

A rise in ventilation will lead to loss of carbon dioxide in the body. Hyperventilation induced hypocapnia can cause respiratory alkalosis. Due to this phenomenon, an SpO2 decrease in NH after 60 s starts to flatten resulting in higher SpO2 readings at the end of exposure (Malle et al., 2013). This is why subjects with lower VE have lower SpO2 readings before the recognition of hypoxia symptoms. Based on the results of this study, hyperventilation during hypoxia has a cumulative negative effect on ILS flight performance.

One weakness in our study was the limited number of subjects. On the other hand, all the subjects had similar flight experience and this was their first simulator hypoxia training ever. Unfortunately we were not able to measure the end-tidal CO2 from masks which resulted in a lack of hypocapnia documentation, but Stepanek et al. (2014) have reported a P_ET_CO2 decrease from 36.2 ± 5.5 mmHg to 30.7 ± 3.2 mmHg after 3 min on 8% oxygen. Additionally, nitrogen kinetics will change with normobaric hypoxic gases. However, gradient between alveolar and arterial nitrogen partial pressure is greater in hypobaric hypoxia compared to our method.

Hyperventilation is one of the reasons for non-pressure hypoxia-like physiological episodes (PE) in-flight. The USAF have reported 73 hypoxia-like symptoms including 4 cases with F-22A and 7 cases with F-35A during flight year of 2019. It is likely that some of the reported PEs are caused by hyperventilation symptoms (Connolly et al., 2021) recognized because of mandatory hypoxia training in military aviation.

In conclusion, hyperventilation during normobaric hypoxia had negative, long-lasting effects on the ILS flight performance. Hypoxia training should include training to regain conscious normal breathing rate and depth after hypoxia emergency procedures. More research is needed to understand the complicated relationship between hypoxia, hyperventilation, hypocapnia and flight performance.

## Data Availability

The raw data supporting the conclusions of this article will be made available by the authors, without undue reservation.
